# Supplementary material for: Realization of high-efficiency fluorescent organic light-emitting diodes with low driving voltage
Source: Nat Commun. 2019 May 24;10:2305. doi: 10.1038/s41467-019-10260-7 (PMC6534605; doi:10.1038/s41467-019-10260-7)
Supplement: Supplementary file 1 — Supplementary Information [file 41467_2019_10260_MOESM1_ESM.docx]

**Supplementary Information**

Realization of High Efficiency Fluorescent Organic Light-Emitting Diodes with Low Driving Voltage

Saleh et al.

**Supplementary Figure 1** Overlap between absorption of BCzVBi and PL emission of CzPA, indicates spectral overlap for Förster energy transfer from singlet state of CzPA to the singlet state of BCzVBi.


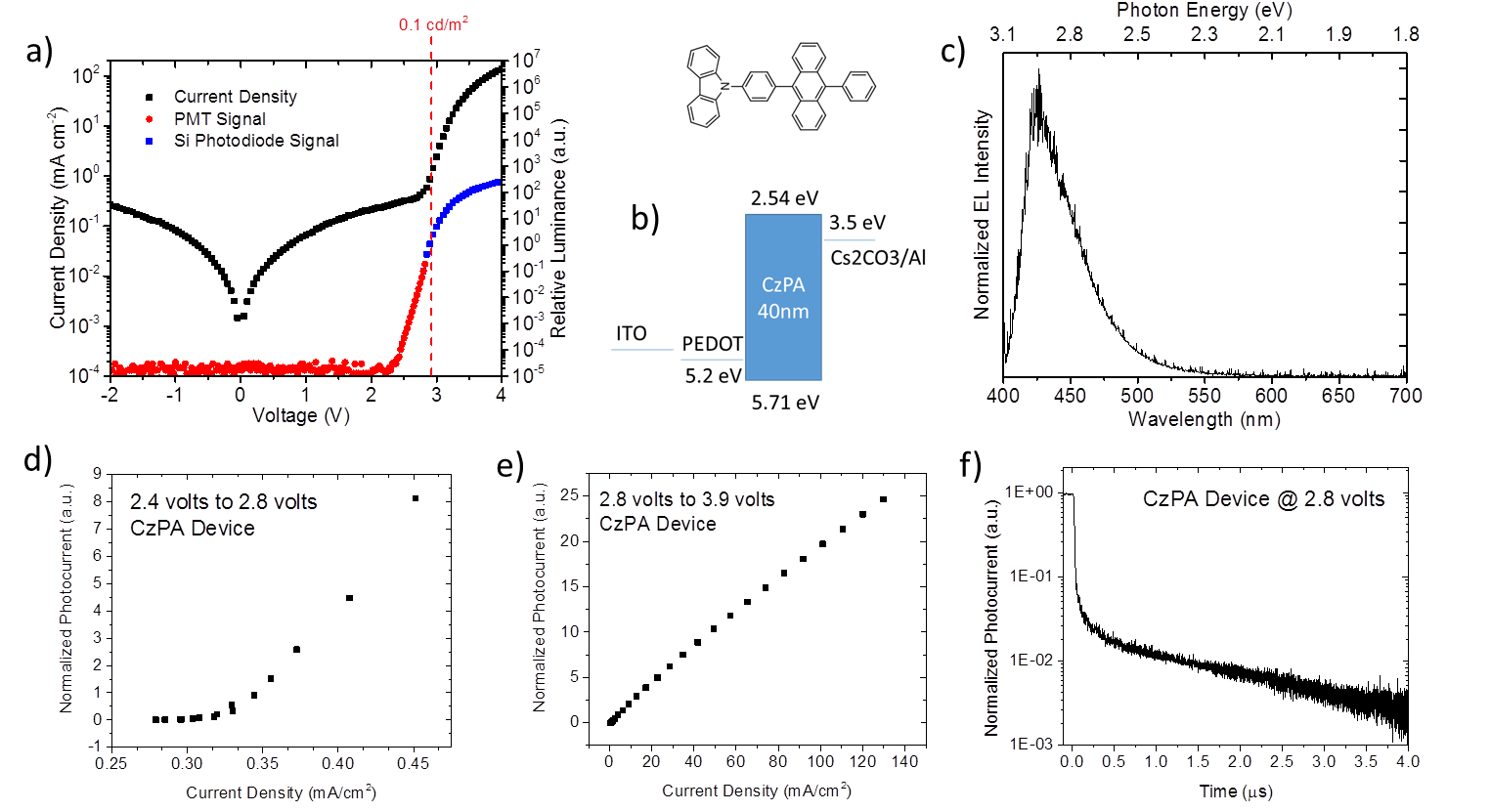


**Supplementary Figure 2** a) Current density and relative luminance versus voltage. b) Molecular structure of CzPA and the energy level diagram of the corresponding single layer device. c) EL spectrum. d) Photocurrent vs. current density in the low-voltage region and e) in the high-voltage region. f) Transient EL dynamics of the device after turn-off.


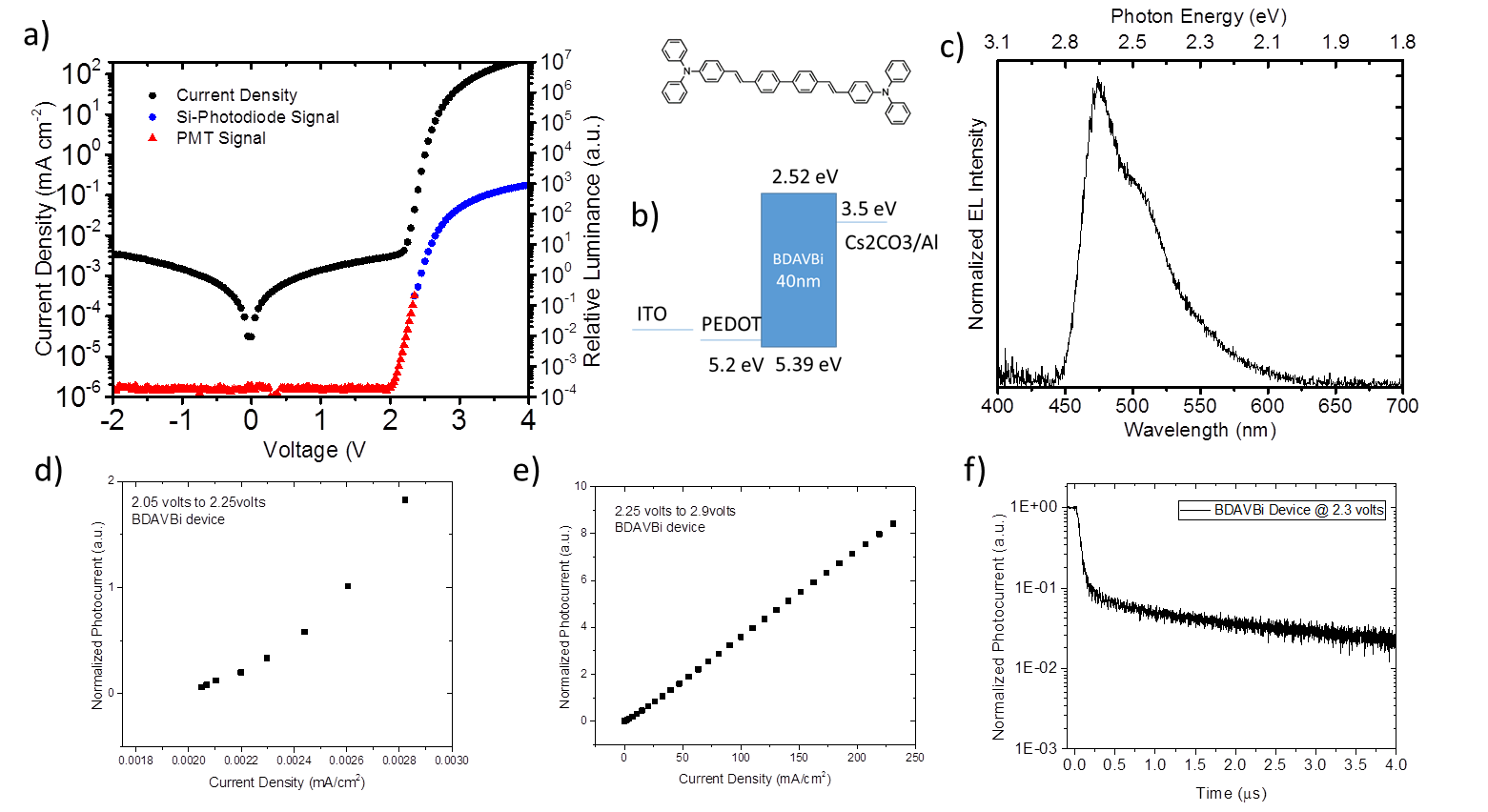


**Supplementary Figure 3** a) Current density and relative luminance versus voltage. b) Molecular structure of BDAVBi and the energy level diagram of the corresponding single layer device. c) EL spectrum. d) Photocurrent vs. current density in the low-voltage region and e) in the high-voltage region. f) Transient EL dynamics of the device after turn-off.


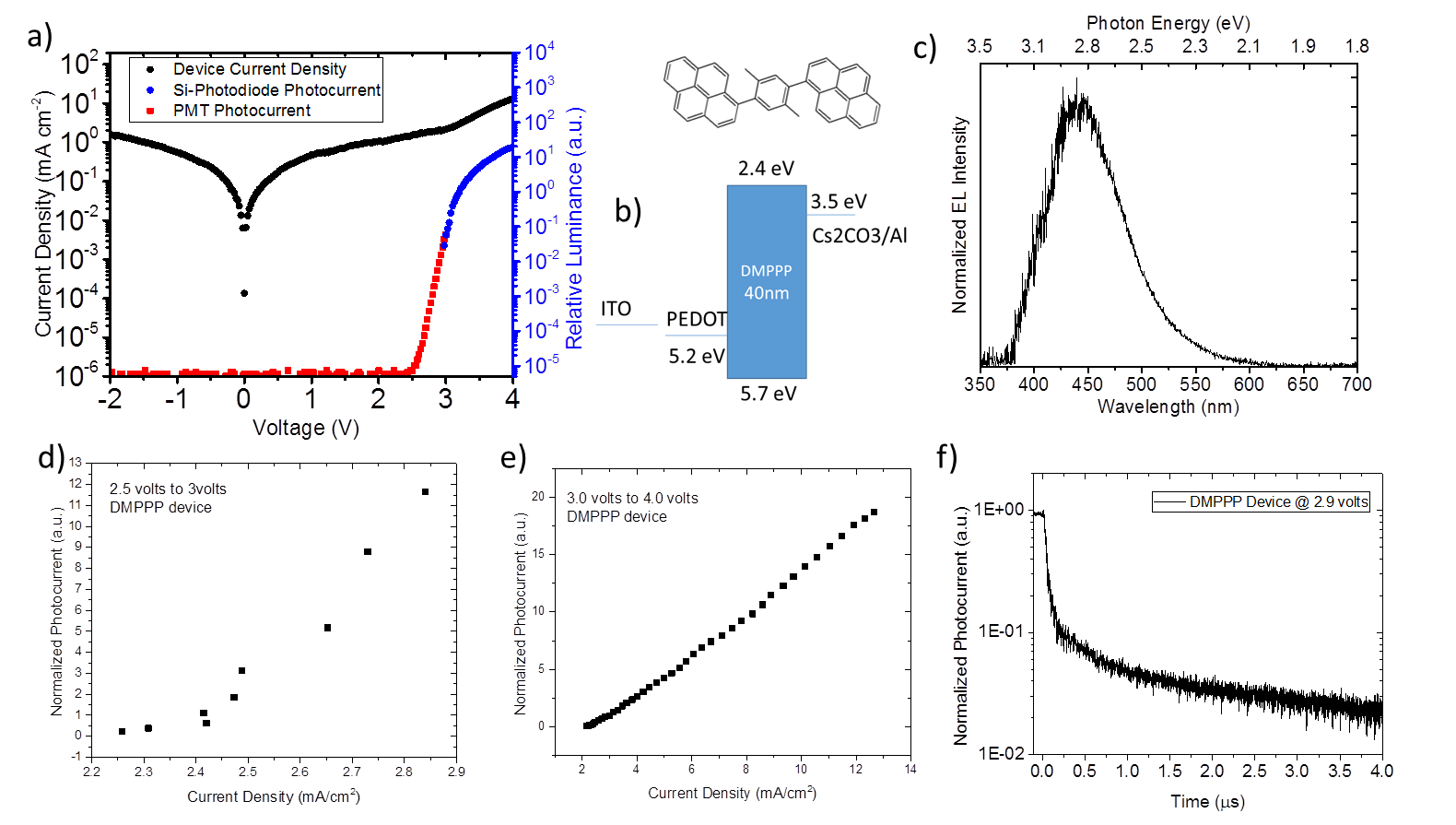


**Supplementary Figure 4** a) Current density and relative luminance versus voltage. b) Molecular structure of DMPPP and the energy level diagram of the corresponding single layer device. c) EL spectrum. d) Photocurrent vs. current density in the low-voltage region and e) in the high-voltage region. f) Transient EL dynamics of the device after turn-off.


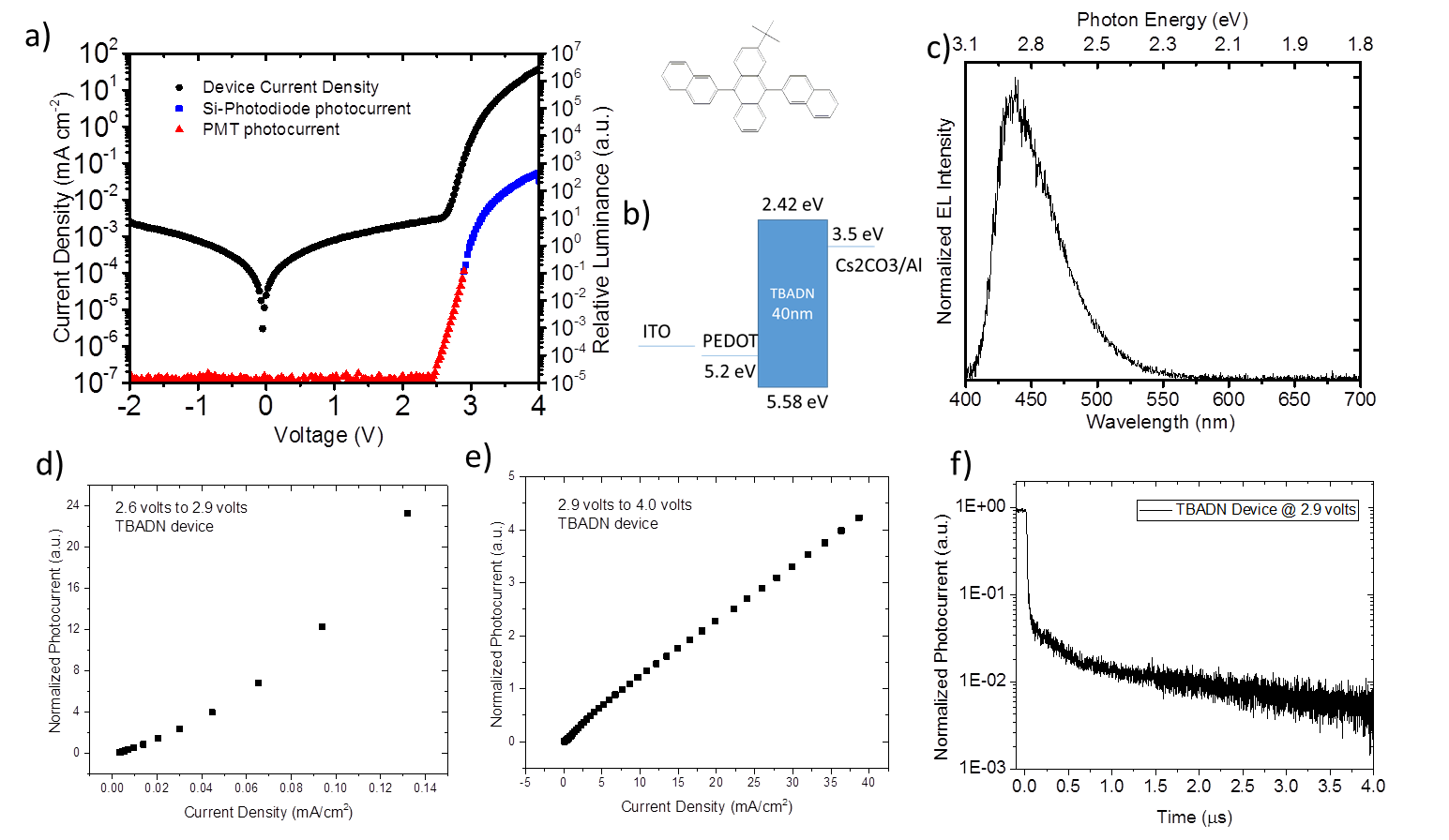


**Supplementary Figure 5** a) Current density and relative luminance versus voltage. b) Molecular structure of TBADN and the energy level diagram of the corresponding single layer device. c) EL spectrum. d) Photocurrent vs. current density in the low-voltage region and e) in the high-voltage region. f) Transient EL dynamics of the device after turn-off.

**Supplementary Table 1** Materials studied in single-layer OLEDs. The OLED structure for all the devices is ITO / PEDOT (30 nm) / Material (40 nm) / Cs_2_CO_3_ (1 nm) / Aluminum (100 nm), except for rubrene. Rubrene device structure was ITO / PEDOT (30 nm) / Rubrene (40 nm) / Aluminum (100 nm).

| Material | First Triplet Excited State^1^ (T_1_)  (eV) | HOMO Level from Vacuum^2^  (eV) | Hole  Injection  Barrier from PEDOT  (eV) | T_1_ + Hole  Injection  Barrier  (eV) | Voltage  at EL  Onset  (V) | HOMO-LUMO gap^3^  (eV) |
| --- | --- | --- | --- | --- | --- | --- |
| BCzVBi | 1.94 | 5.38 | 0.18 | 2.12 | 2.10 | 3.03 |
| CzPA | 1.77 | 5.71 | 0.51 | 2.28 | 2.35 | 3.17 |
| DMPPP | 2.06 | 5.70 | 0.50 | 2.56 | 2.50 | 3.30 |
| TBADN | 1.82 | 5.58 | 0.38 | 2.20 | 2.50 | 3.16 |
| BDAVBi | 1.86 | 5.39 | 0.19 | 2.05 | 2.00 V | 2.86 |
| Rubrene | 1.14 | 5.24 | 0.04 | 1.18 | 1.10 V | 2.28 |

^1^ Triplet state energies of the materials were measured using phosphorescence, as shown in Figure S10. ^2^ HOMO levels were measured using cyclic voltammetry as describes in the Experimental Section. ^3^HOMO-LUMO gaps (bandgap) were estimated from solution-phase absorption and emission spectra, as presented in Figure S11.


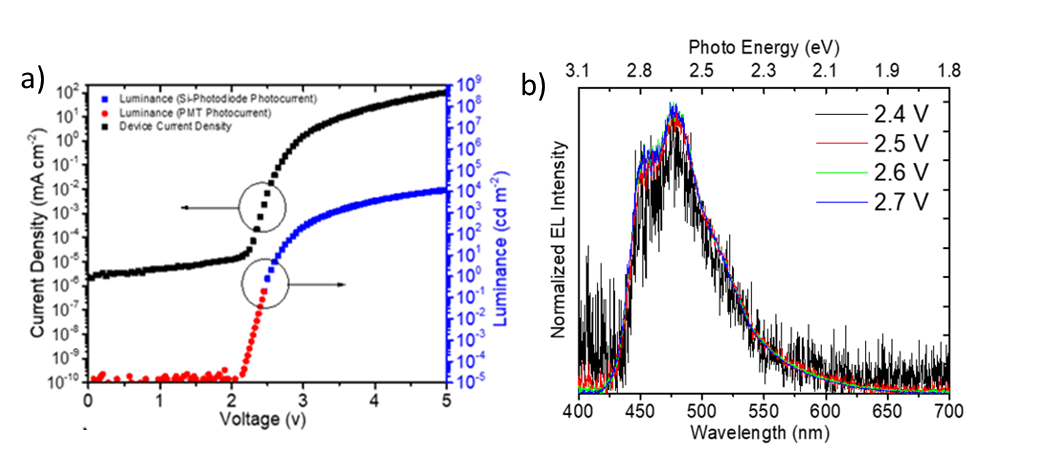


**Supplementary Figure 6** a) Current density and luminance (both PMT and photodiode) versus voltage of the multi-layer device. b) EL spectra at different voltage.


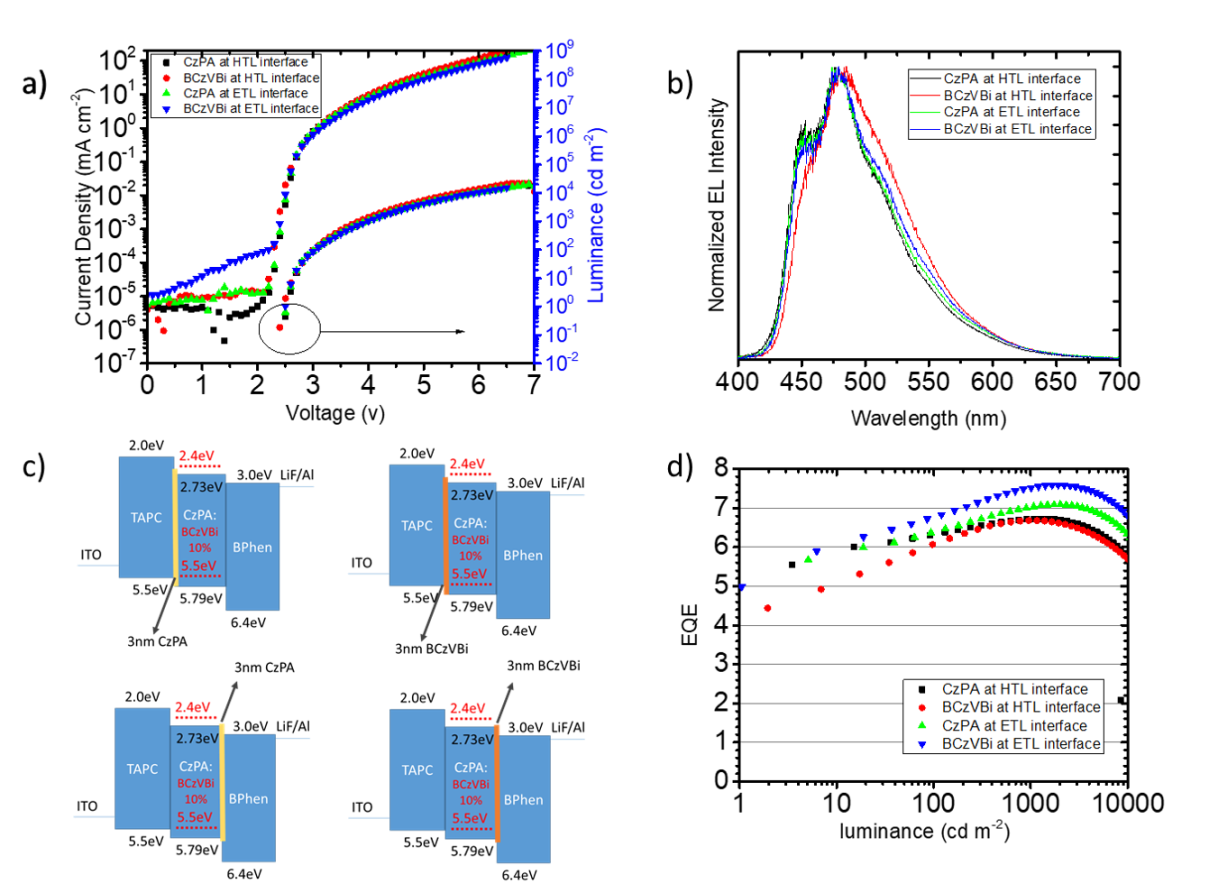


**Supplementary Figure 7** Devices with different HTL or ETL to investigate if there is exciplex formation. a) Current density and luminance versus voltage characteristics. b) EL spectra. c) Device structures. d) EQE-luminance characteristics.


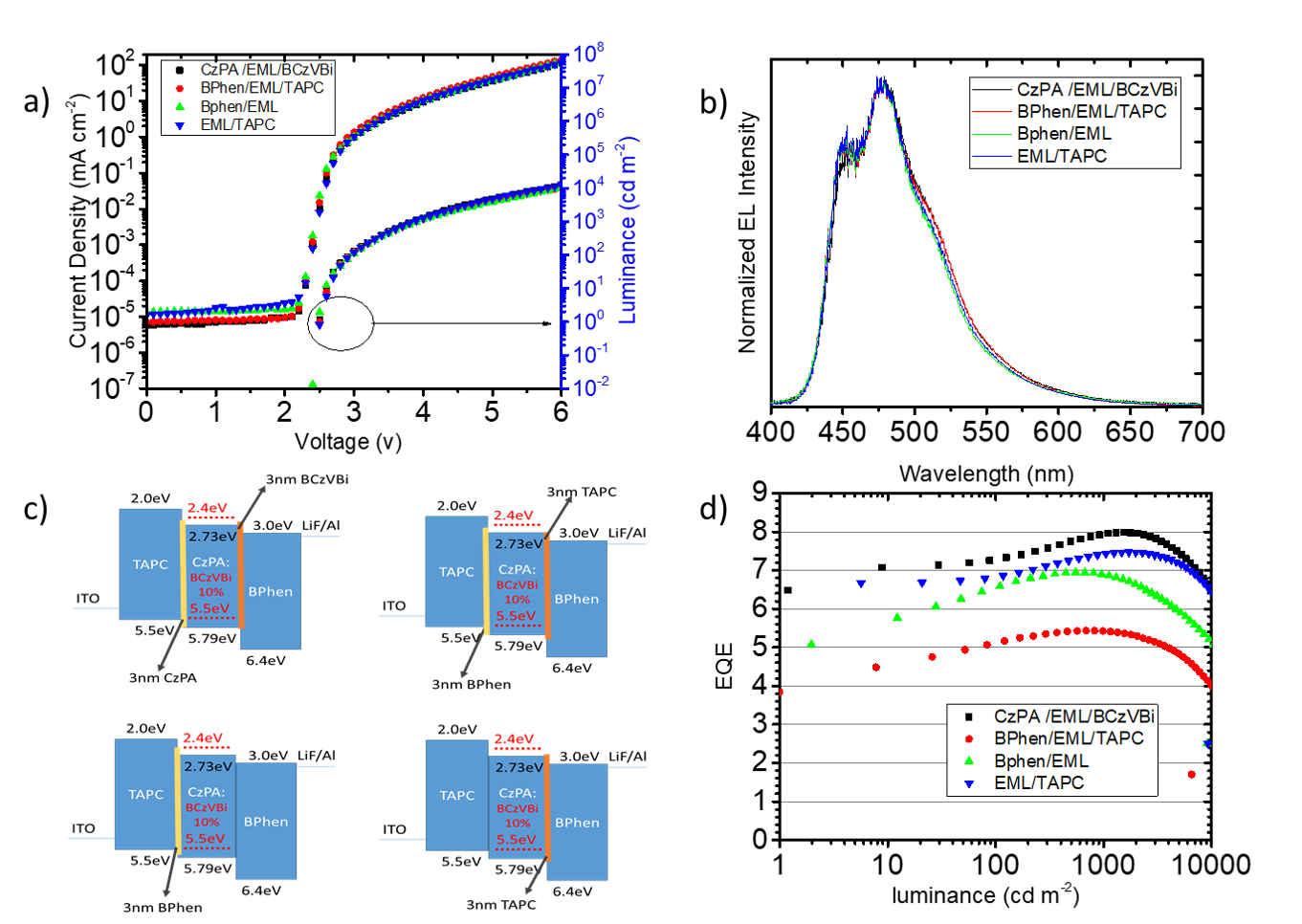


**Supplementary Figure 8**  Charge balance optimization run in consideration of hole and electron injection interface. a) Current density and luminance versus voltage characteristics. b) EL spectra. c) Device structures. d) EQE-luminance characteristics.


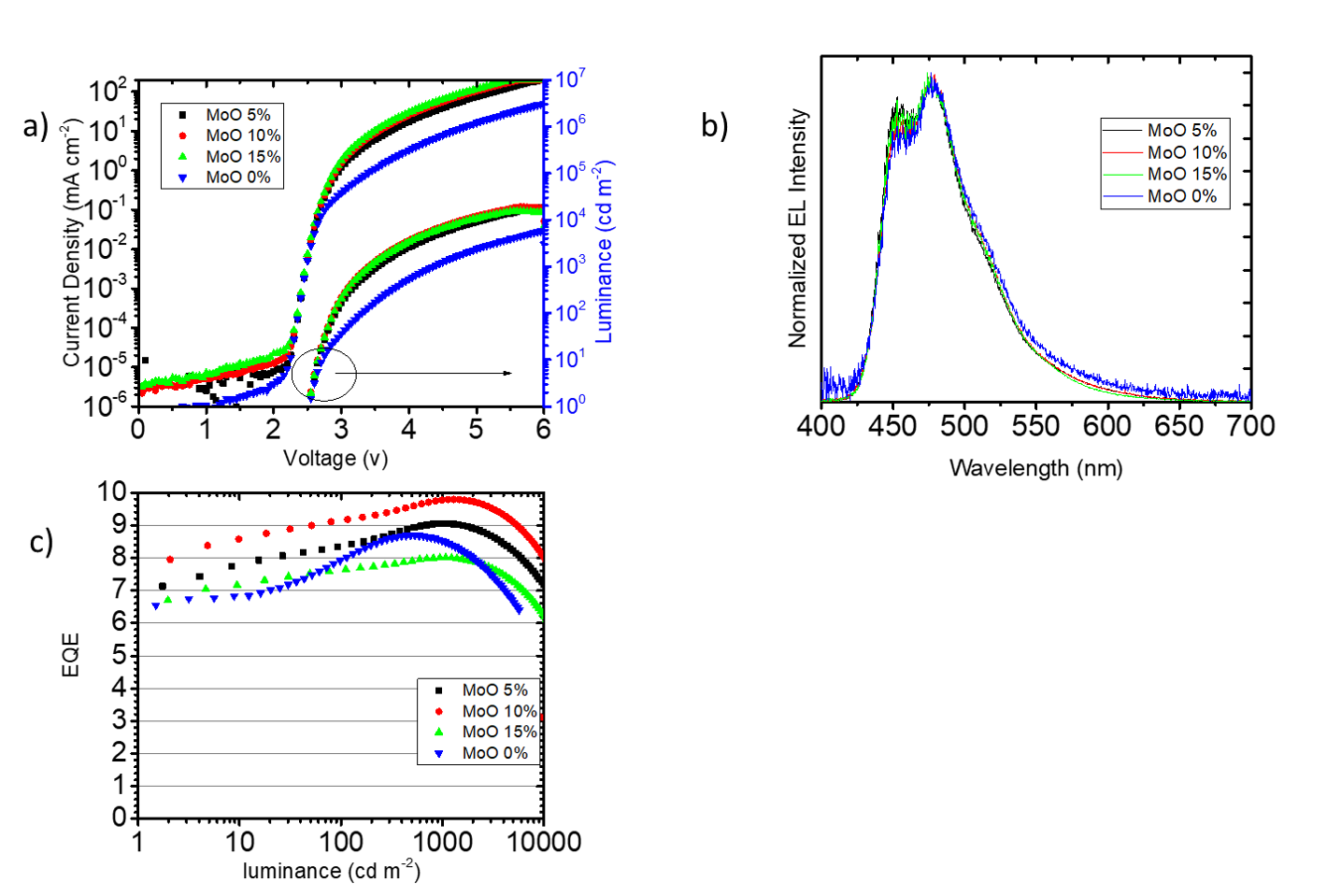


**Supplementary Figure 9**  Charge balance optimization run in consideration of MoO_3_ doping ratio in TAPC. a) Current density and uminance versus voltage characteristics. b) EL spectra. c) EQE-luminance characteristics. Device structure is ITO (50 nm) / TAPC:MoO_3_ 1:x (30 nm) / TAPC (20 nm) / CzPA (3 nm) / CzPA:BCzVBi 1:0.1 (30 nm) / BCzVBi (3 nm) / BPhen (30 nm) / Cs_2_CO_3_ (1 nm) / Aluminum (100 nm).


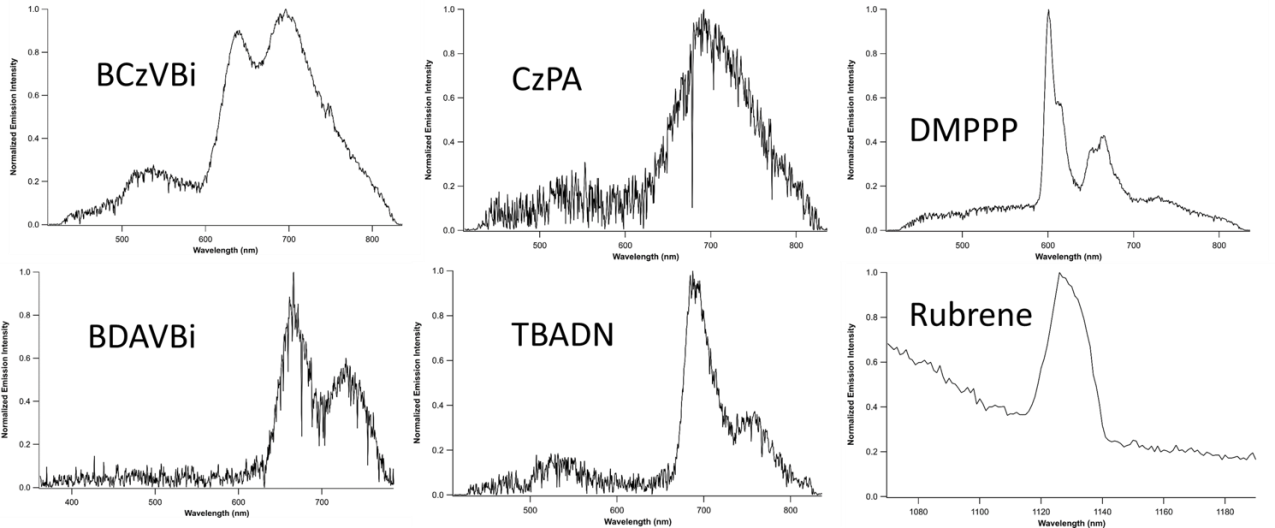


**Supplementary Figure 10** Phosphorescence spectra of the molecules studied in this work recorded at 77 K in 40:40:20 toluene:dichloromethane:iodobenzene. Molecular triplet energies were experimentally estimated via the peak in the respective phosphorescence spectra.


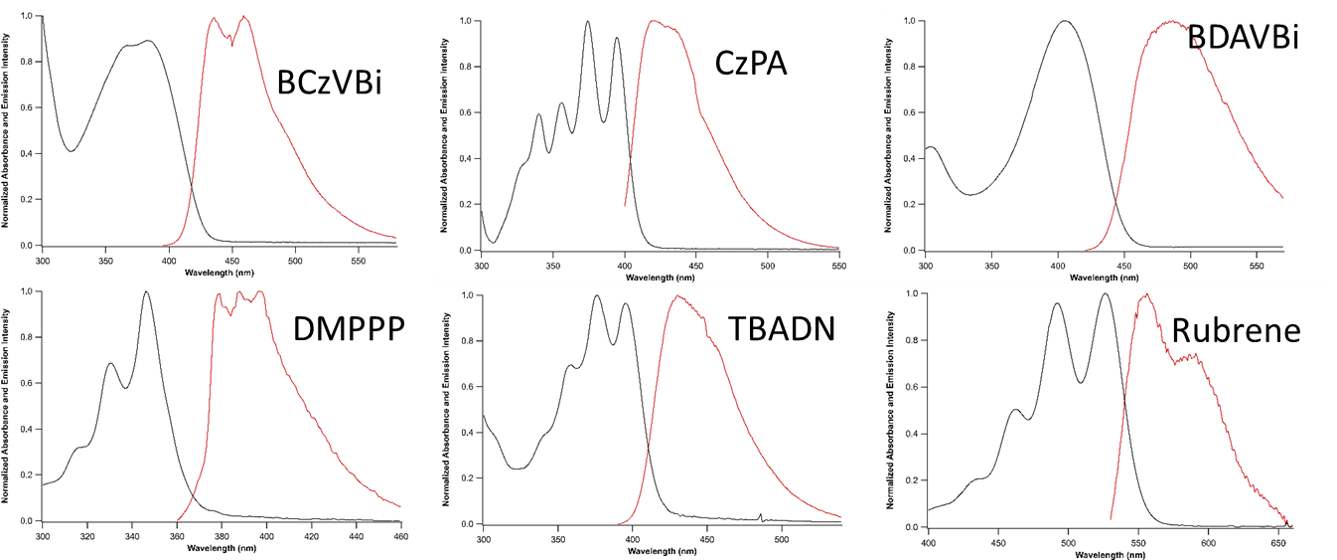


**Supplementary Figure 11** Fluorescence (red) and absorption (black) spectra of the molecules used in this study measured in toluene. Molecular singlet energies were estimated from the E_00_ determined from the overlap in the respective absorption and emission spectra.
